# Health Disparities and Cancer: Racial Disparities in Cancer Mortality in the United States, 2000–2010

**DOI:** 10.3389/fpubh.2015.00051

**Published:** 2015-04-15

**Authors:** Eileen B. O’Keefe, Jeremy P. Meltzer, Traci N. Bethea

**Affiliations:** ^1^Department of Health Sciences, Boston University, Boston, MA, USA; ^2^Slone Epidemiology Center, Boston University, Boston, MA, USA

**Keywords:** cancer mortality, socioeconomic status, race/ethnicity, disparities, affordable care act

## Abstract

Declining cancer incidence and mortality rates in the United States (U.S.) have continued through the first decade of the twenty-first century. Reductions in tobacco use, greater uptake of prevention measures, adoption of early detection methods, and improved treatments have resulted in improved outcomes for both men and women. However, Black Americans continue to have the higher cancer mortality rates and shorter survival times. This review discusses and compares the cancer mortality rates and mortality trends for Blacks and Whites. The complex relationship between socioeconomic status and race and its contribution to racial cancer disparities is discussed. Based on current trends and the potential and limitations of the patient protection and affordable care act with its mandate to reduce health care inequities, future trends, and challenges in cancer mortality disparities in the U.S. are explored.

## Introduction

Cancer mortality rates in the United States (U.S.) began to decline in the early 1990s, following favorable trends in cancer risk factor reduction, such as reduced tobacco smoking among adults, more widespread cancer screening and testing, and improved cancer therapies (1). This decline has continued through the first decade of the twenty-first century. However, Black-Americans continue to have the highest cancer mortality and shortest survival time among racial or ethnic groups, with the exception of American-Indian/Alaska-Natives. The significant Black–White disparity highlights national trends and is the focus of this review.

Definition and measurement of health disparities itself has also undergone a significant evolution over the past two decades. There is substantial evidence regarding racial/ethnic and socioeconomic disparities in health-related behaviors, health status, and health outcomes. Any assessment of racial disparities is compounded by the fact that negative socioeconomic factors disproportionately impact Black-Americans. Although disparities exist throughout the continuum of cancer epidemiology, from exposures through outcomes, this review concentrates on cancer outcomes. Controlling for cancer stage, cancer diagnosis presents the opportunity for a “level-playing field” from which subsequent cancer outcomes across population groups may be compared. Medical care, specifically appropriate cancer treatments, driven by cancer biology, can yield improved outcomes for specific cancers among some racial and ethnic groups. However, overall cancer deaths continue to be significantly higher among Black-Americans than White-Americans (2). This review focuses on lung, colorectal, female breast, and prostate cancer – cancers with high incidence rates (lung, colon/rectum, female breast, and prostate) and high mortality rates (lung and colon/rectum) using data from the National Cancer Institute’s Surveillance, Epidemiology, and End Results (SEER) program, and on factors contributing to cancer disparities.

## Defining a Disparity

Socioeconomic and racial/ethnic disparities in health have been recognized and documented in U.S. and the United Kingdom for decades. The landmark Whitehall Study in Britain, begun in 1967, demonstrated an inverse relationship of social class and disease mortality (3, 4). Similar reports emerged in the U.S., highlighting differences in disease incidence, survival, and mortality rates based on socioeconomic status and race/ethnicity. These health disparities also exist for cancer (5, 6). Despite the consensus that social disparities in cancer exist, there has not been consensus on disparity definition and measurement, and whether disparities should be measured at the individual or ecological level (7–10). In fact, the U.S. has experienced an evolution in vocabulary to define and describe health disparities (Table 1). Prior to 2005, the terms *health equity* and *health inequity* were avoided in favor of *health disparity* (11). More recent federal and international documents, however, embrace these terms. Notably, Healthy People 2020 contains health equity as one of its overarching goals (12). The World Health Organization refers to a health disparity as “the unfair and avoidable differences in health status seen within and between countries.” (13) This concept is vitally important. Though the term health disparities implies a sense of injustice, explicit inclusion of the terms equity, inequity, and injustice, recognizes that social injustice contributes to health disparities.

**Table 1:** **Defining a disparity**.

Agency	Term used	Definition
U.S. Department Health and Human Services^a^^,^^b^	Health disparities	*Healthy People 2010*: differences in the incidence, prevalence, mortality and burden of diseases, and other adverse health conditions that exist among specific population groups in the United States
	Health disparity	*Healthy People 2020*: a particular type of health difference that is closely linked with social, economic, and/or environmental disadvantage
	Health equity	*Health People 2020*: attainment of the highest level of health for all people. Achieving health equity requires valuing everyone equally with focused and ongoing societal efforts to address avoidable inequalities
U.S. Department of Health and Human Services Agency for Healthcare Research and Quality (AHRQ)^c^	Health care disparities	Differences or gaps in care experienced by one population compared with another … within the scope of health care delivery, these disparities may be due to differences in access to care, provider biases, poor provider-patient communication, poor health literacy, or other factors
Institute of Medicine (IOM)^d^	Disparities	Racial or ethnic differences in the quality of health care that are not due to access-related factors or clinical needs, preferences, and appropriateness of intervention
U.S. National Institutes of Health National Cancer Institute (NCI)^e^	Cancer health disparities	Differences in the incidence, prevalence, mortality, and burden of cancer and related adverse health conditions that exist among specific population groups in the United States
World Health Organization (WHO)^f^	Health inequities	Avoidable inequalities in health between groups of people within countries and between countries that arise from inequalities within and between societies

*^a^U.S. Department of Health and Human Services (2000). *Healthy People 2010: Understanding and Improving Health 2nd Ed*. Washington, DC, USA: U.S. Government Printing Office*.

*^b^U.S. Department of Health and Human Services (2014). Health People 2020: *Title* Washington, DC, USA: U.S. Government Printing Office. Accessed August 7, 2014 at: http://www.healthypeople.gov/2020/about/disparitiesAbout.aspx*.

*^c^Agency for Healthcare Research and Quality, *2009 National Healthcare Disparities Report* (Rockville, MD, USA: AHRQ 2009) Accessed August 14, 2014 at http://archive.ahrq.gov/research/findings/nhqrdr/nhdr09/index.html*.

*^d^Smedley BD, Stith AY, Nelson, eds, *Unequal Treatment: Confronting Racial and Ethnic Disparities in Health Care* (Washington National Academies Press) (2003), p. 3–4*.

*^e^National Cancer Institute Accessed August 27, 2014 http://crchd.cancer.gov/about/defined.html*.

*^f^World Health Organization. Social determinants of health Accessed August 27, 2014 http://www.who.int/topics/social_determinants/en/ or http://www.who.int/social_determinants/thecommission/finalreport/key_concepts/en/*.

How society views race and ethnicity in this context is critical to the measurement and elimination of disparities. From the perspective of health disparities, race is correctly viewed as a social construct (14). Race is a “social classification, based on phenotype, and is a marker for social factors, which influence health” (15), primarily socioeconomic status. Though distinctions are made between race and ethnicity, both are social constructs and, from the perspective of health disparities, the terms overlap and can be viewed in combination as race/ethnicity. The decision on which variables to include and adjust in statistical models to quantify racial and ethnic health disparities impacts the resultant magnitude of the disparity. Variables such as level of education, income, and even diet, which are closely linked to the environment and social context in which individuals live, may contribute to disparities and may more correctly be excluded from the statistical model lest they serve to minimize or hide a “true” disparity (15). Agreement on whether or not to exclude variables, which are socially determined from statistical modeling used to measure racial and ethnic disparities would serve to generate results that are comparable across studies and could result in a more accurate picture of the magnitude of racial and ethnic disparities in the U.S. over time.

SES measures, such as level of education, income, and deprivation are not consistently collected in public health surveillance systems. This inconsistency adds a further complexity to disparities assessment. When data are available, these data are often subjective, provided by self-report, and subject to potential inaccuracies. Where individual measures are lacking or as an alternate, ecological SES measures have demonstrated value. Two basic issues lie at the heart of ecological SES measures: which socioeconomic measures to include and at what geographic level (10, 16–18). Though ecological measures provide a level of socioeconomic context, the utility of these variables are limited by ecological bias in that measures at the group level may not reflect those at the individual level. The Public Health Geocoding Project (8, 19) reports that data collected for block groups and census tracts, with an average 1,000 and 4,000 inhabitants, respectively, detect SES gradients whereas data for zip codes, with an average 30,000 inhabitants, fail to detect these differences. Data measures on economic poverty were strongest at detecting gradients, which were not observed with ecological measures of education. Thus, block group or census tract measures, such as percent of population below the poverty level, may provide a consistent and measurable SES marker at the smaller ecological levels (19).

Another contributor to SES-related disparities in cancer mortality is residential segregation. In the U.S., residential segregation remains highest for Blacks, compared to other racial/ethnic minorities (20). Residential segregation concentrates Blacks in poorer quality, more economically deprived neighborhoods than Whites (21–23). Additionally, residential segregation restricts access to quality health care, fresh produce, recreational facilities, and economic mobility (24–29), which may influence risk of cancer mortality. Studies have reported the impact of racial residential segregation on cancer health care and cancer mortality using Census tract data (30–33). In the U.S., lung cancer mortality among Blacks is higher than among Whites, but is highest among Blacks living in the most segregated neighborhoods (30). Blacks residing in the neighborhoods, which were most racially segregated have a 10% higher lung cancer mortality rate compared with those who reside in the least segregated neighborhoods, a disparity that persists after adjustment for socioeconomic status. This association is strongly supported by the demonstration that an incremental increase in racial segregation is associated with a corresponding increase in lung cancer mortality among blacks. Studies examining racial residential segregation and breast cancer care (31–33) indicate significant disparities in breast cancer care by race. Overall, Blacks are more likely to present with late stage cancer diagnosis. Higher racial segregation is associated with lower mammography access and late stage cancer diagnosis. Black residential segregation is estimated to account for 8.9% of this difference in health cancer care. Inclusion of measures of residential segregation is an important and relevant variable in assessment of cancer disparities. This review focuses on characteristics of socioeconomic and structural (e.g., economic resources, societal attitudes) factors that contribute to disparities in cancer outcomes by race/ethnicity.

## Methodology

The literature search was completed using MEDLINE and Google Scholar. Search terms included combinations of “cancer,” “disparities,” “race,” “ethnicity,” “socioeconomic,” and “mortality.” Reference lists of comprehensive review articles were examined for relevant articles not available in MEDLINE and Google Scholar searches. This review focuses on recent mortality trends, and the literature search was generally limited to articles published in 2004 or later. Reported cancer incidence and mortality data for the years 2000 through 2010 were extracted from the SEER program of the National Cancer Institute. The SEER program reports incidence, prevalence, mortality, and survival data dating back to 1969 from reporting states and municipalities for some catchment areas. Incidence rates were based on data from the SEER 18 database for the years 2000–2011 (34). Mortality rates were taken from the mortality – all COD SEER database for the years 1969–2010 (35). Cancer mortality disparity ratios were calculated using percent differences between Black and White mortality by site. These were graphed to include trend lines based on 10-year changes in mortality. The discussion in this review thus focuses on characteristics of socioeconomic and structural factors that contribute to the resulting disparity ratios in cancer outcomes by race/ethnicity.

## Overall Cancer Mortality

Between 2000 and 2010, overall cancer incidence was higher among Black men compared to White men and higher among White women compared to Black women (34). Cancer mortality over the same time period was higher for both Black men and women compared to White men and women (35). Between 2005 and 2009, the overall age-adjusted mortality among Black men was 288.3/100,000 compared to 216.7/100,000 for White men. Despite lower cancer incidence among White women, age-adjusted mortality was higher among Black women compared to White women (180.6/100,000 compared to 155.0/100,000) (35). These contrasting statistics indicate that there are factors, which affect the outcomes for Black and White Americans after a cancer has been detected and diagnosed. This review focuses on potential contributors to these differences, with emphasis on the four sites: lung, colon/rectum, female breast, and prostate.

**Figure 1:**
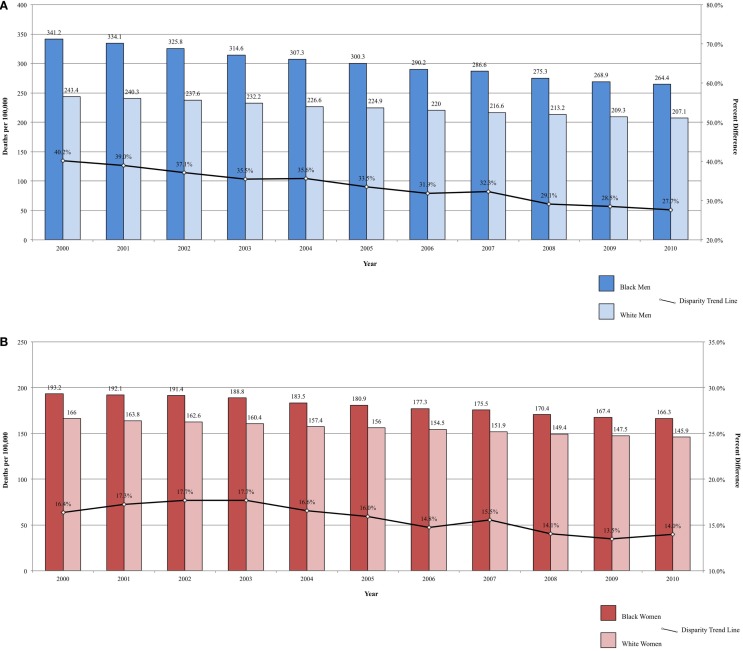
**(A)** All-site cancer mortality among men. United States 2000–2010 and disparity ratio trend. **(B)** All-site cancer mortality among women, United States 2000–2010 and disparity ratio trend.

Fixed-interval trends between 2000 and 2009 show some encouraging findings. For Black men, all-site incidence and mortality are declining at a faster rate than among White males (Figure 1A). Lung cancer incidence and mortality and prostate cancer mortality were also declining faster among Black men. For the same time period, all-site mortality declined faster among Black women than among White women (Figure 1B). Lung cancer incidence and mortality, as well as cervical cancer incidence and mortality, declined faster among Black females. The rate of these declines is of interest because, if sustained, these trends suggest that racial disparities may continue to narrow and could potentially be eliminated over time.

## Lung Cancer

Lung cancer is the leading cause of cancer mortality in the U.S. The downwards trend in tobacco smoking among adults following the first Surgeon General’s report on tobacco and health in 1960s has had the greatest impact on overall cancer rates, particularly cancers of the lung and bronchus (1). Reflecting patterns in cigarette smoking, in which cessation has been more pronounced among men, with the risk of lung cancer reducing approximately linearly until around 20 years after cessation (1). Whites and Blacks have significantly higher smoking prevalence than other racial/ethnic groups in the U.S. For example, the 16.2% smoking prevalence among Hispanics is significantly lower than both Blacks and Whites, as is lung cancer incidence and mortality (34–37).

Since the late 1970s, there has been a steep decrease in initiation of cigarette smoking among Black men, resulting in a narrowing gap in lung cancer incidence rates between White and Black men under the age of 40 (38). Between 2005 and 2010, current smoking prevalence for Black men over the age of 18 decreased from 26.7 to 24.8%, a greater drop than for White men over the same time period: a smaller decrease from 24.0 to 22.6% (36). Despite the steady decline in new diagnoses, Black men continue to have a higher lung cancer incidence than White men. White women have consistently higher lung cancer incidence rates than Black women, reflecting historical female racial smoking patterns (35). Both squamous cell and small cell (SCLC) carcinomas are strongly associated with cigarette smoking. The changing prevalence of histologic subtypes over time shows strong evidence of this association between tobacco smoking and lung cancer. Since 1980s, the rates of squamous cell carcinoma have been decreasing, paralleling the decline in smoking prevalence (39). The fraction of lung cancers classified as small cell (SCLC), which are rare among non-smokers, has also been decreasing (40). Between 1986 and 2002, the proportion of lung cancers classified as small cell has decreased from 17.26 to 12.95% (41). As SCLC rates have declined, the proportion of lung cancers that have a weaker association with smoking (e.g., adenocarcinoma) has increased. Between 2004 and 2009, there were higher incidence rates of squamous cell carcinoma and adenocarcinoma among Black men and higher rates of SCLC among White men, suggesting a correlation between the greater prevalence of smoking and smoking-related lung cancers among Black men (39). For women, the histologic incidence rates are not indicative of smoking patterns since this histologic transition is not discernible for women over the same period of time as for men.

For the years 2000–2009, Black men had higher lung cancer mortality compared to White men, but lung cancer mortality rates declined faster in Black men (3.0%/year) and women (1.0%/year) compared with White men (2.2%/year) and women (0.6%/year) (2, 38). Declines among Black and White men were not as great as the declines among Hispanic men (3.3%/year) (37). A morbidity and mortality weekly report on current smoking among adults reports higher prevalence among Black men compared to White men and for White women compared to Black women in surveys conducted in 2005 and 2010, suggesting future trends in lung cancer incidence by race and gender (36).

**Figure 2:**
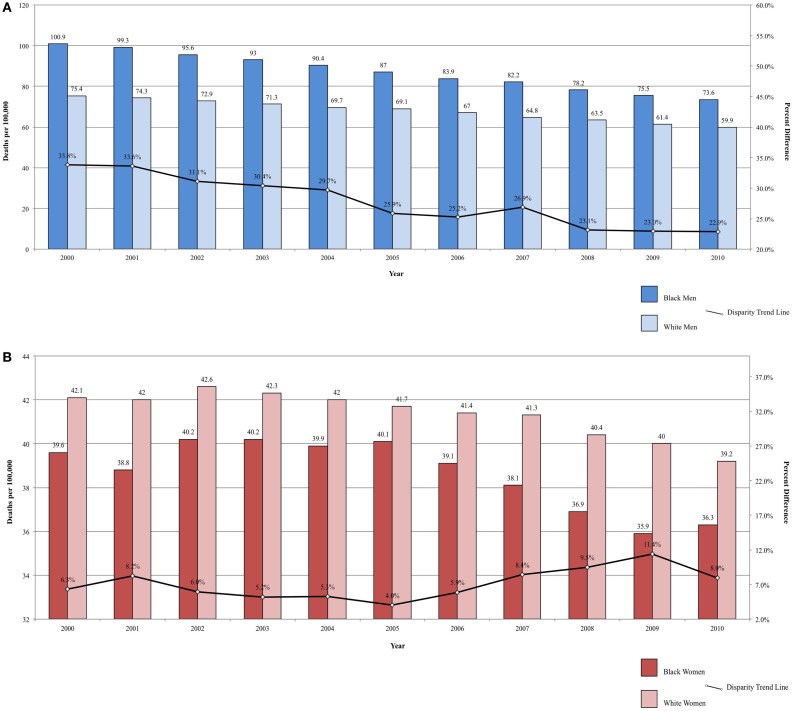
**(A)** Lung cancer mortality among men, United States 2000–2010 and disparity ratio trend. **(B)** Lung cancer mortality among women, United States 2000–2010 and disparity ratio trend.

Between 2000 and 2010, the ratio in lung cancer mortality by race decreased from 33.82 to 22.87% higher among Black men, for an annual disparity decline of 1.09% (Figure 2A). Over the same time period, lung cancer mortality increased from 5.94 to 7.40% higher among White women compared to Black women, for an annual disparity increase of 0.15%/year (Figure 2B). These trends are reflective of changes in smoking prevalence patterns.

Though the disparity in lung cancer death rates between Black and White men has been substantially reduced from a 50% difference in 1990–1992 to a 26% difference in 2005–2009, the 5-year overall relative survival has remained lower in Blacks compared to Whites (13 and 16%, respectively). Disparities in lung cancer mortality can be explained by a number of factors. Blacks are more likely to present at a more advanced stage than Whites (60 vs. 55%) (2). Surgical resection, the most successful treatment, is an option only for localized disease (42) and individuals presenting with advanced disease, have fewer options for successful medical intervention. Thus, Black patients are less likely to have the option of surgical resection, which may contribute to the lower 5-year survival. Further, Blacks are less likely to have surgical resection even when they presenting with localized disease (42). There are lower rates of recommendations for lung cancer surgery in Blacks (67% for Blacks and 71.4% for Whites) and higher refusal rates after surgery was recommended (3.4% for Blacks and 2.0% for Whites) (43).

Survival rates post-treatment also demonstrate racial variations. After treatment for advanced non-small cell lung cancer, 1-year survival was 22% in Black patients and 30% in White patients (44). This finding presents evidence that factors beyond disease stage and access to care affecting lung cancer outcomes. One hypothesized factor is routine access to cancer care before diagnosis, which, if not available, can result in poorer overall patient health at diagnosis (44). In an equal access health care system (the U.S. military health system), where routine access to cancer care is comparable for Blacks and Whites, lung cancer survival was the same for Black and White patients (45).

Studies have shown an association between lower socioeconomic status and higher risks for cancer, including lung cancer, and a greater likelihood of presenting at a more advanced disease stage. This relation is indicative of a complex underlying relationship between race/ethnicity, socioeconomic status, and lung cancer outcomes. For example, Black-Americans are almost three times more likely to live in poverty than Whites (35 vs. 13%) and poverty is associated with increased incidence of lung cancer and the likelihood of presenting at a more advanced (non-localized) stage, which is related to poorer prognosis and survival (10, 46). Adding to the complexity, individuals with a lower SES, indicated by both poverty status and education attainment, are also more likely to be current cigarette smokers (36).

The strongest risk factor for lung cancer, cigarette smoking, and the most important determinant of lung cancer mortality, advanced stage at diagnosis, are both more prevalent in Black-Americans compared to White-Americans. These differences are related to the higher incidence and poorer survival in Black men. Although the difference in cigarette smoking between Black and White men is decreasing, other factors beyond individual smoking behavior persist, contributing to the differences in lung cancer stage at diagnosis and outcomes between Black and White men. These contributors include differential access to care, more advanced disease at diagnosis, differences in treatment recommendations, and higher rates of surgery refusal. For women, the higher smoking prevalence among White women contributes to higher lung cancer incidence compared to Black women. The mortality disparity between Black and White women with greater mortality rates among Black women likely reflects the same societal differences contributing to the disparity seen in men. The continuing decline in tobacco use in the U.S. will result in downward trends in lung cancer mortality for both genders and the greater cessation rates among Black men suggest that the racial disparity ratio will narrow further and possibly disappear in men. Defining and addressing societal factors will be necessary to eliminate lung cancer survival disparities.

## Female Breast Cancer

In the U.S., breast cancer has the highest cancer rate of new cases among women and is the second leading cause of cancer death in women. Between 2000 and 2009, breast cancer incidence rates increased slightly (0.7%/year) among Black women and decreased (1.0%/year) among White women (47). Prior to 2002, hormone replacement therapy (HRT) was routinely recommended for premenopausal and postmenopausal women. Evidence from the Women’s Health Initiative and other studies reported that HRT use may actually increase risk of breast cancer, as well as heart disease and stroke (1, 48). In response to these reports, there has been a steep decline in HRT use in the U.S., which has been followed by a decrease in breast cancer incidence.

**Figure 3:**
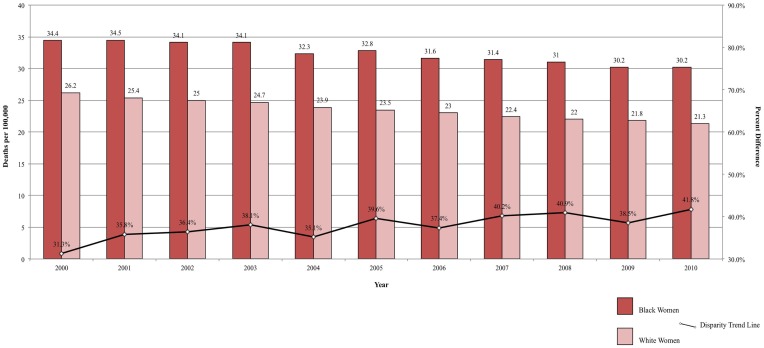
**Breast cancer mortality among women, United States 2000–2010 and disparity ratio trend**.

Although breast cancer mortality has decreased annually by an average of 2%, primarily due to earlier diagnosis and improvements in treatment (49), this decline has been slower in Black women than in White women (47). Breast cancer mortality is higher among Black (50–53), Hispanic (50–52), and Native American (51, 52, 54) women than among White women. For example, between 2002 and 2008, the relative 5-year survival for incident breast cancers was 78% among Black women and 90% among White women (47). The gap in breast cancer mortality between Black and White women is increasing and, between 2000 and 2010, the breast cancer mortality disparity ratio increased from 30.3 to 41.8% (Figure 3). In White women, 5% of breast cancers are detected at an advanced stage, compared to 8% of breast cancers in Black women (47). The difference in breast cancer mortality may be due to more advanced stage at diagnosis and poorer stage-specific survival among Black women (47). The later stage at diagnosis among black women has been attributed to lower frequency of mammograms, greater intervals of time between mammograms, and less consistent follow-up of suspicious mammogram results (47). Even so, across all stages of diagnosis, Black women have poorer survival than White women. A potential explanation is disparate access to high-quality treatment for Black women (47). Additionally, Black women are more likely to have longer delay from diagnosis until treatment (55), are less likely to complete treatment appropriate for the tumor characteristics (56), and are more likely to refuse treatment (57), compared to White women.

The observed difference in breast cancer mortality between Black and White women may also be influenced by more aggressive tumor characteristics in Black women (58). Breast cancers are often classified by at least three immunohistochemical markers – estrogen receptor (ER), progesterone receptor (PR), human epidermal growth factor receptor (HER2). Hormone-negative tumors have been associated with poorer survival (59–62), which may be due to the lack of successful therapies for ER-negative tumors (63, 64). Hormone-negative tumors are more prevalent in Black (52, 60, 62, 65) and Hispanic (52, 62, 66–68) women, compared to White women. In a study of women with tumors that are negative for ER, PR, and HER2, Black women experienced double the risk of mortality compared to White women (60).

Socioeconomic status also correlates with disparities in breast cancer mortality. For breast cancer, like many other cancers, living in a geographic area with high poverty and/or low educational attainment is associated with poorer outcomes (54). The association between low area SES and poorer survival has been observed for Black (69), Hispanic (61, 69), Asian (69), and White (70) breast cancer patients. Low area-level SES is also associated with hormone-negative breast cancer in Black, Hispanic, Asian/Pacific Islander, and White women (62, 71), which may influence disparities in mortality. Individual-level SES factors are inversely associated with mortality in breast cancer patients (72, 73). Among a cohort of White breast cancer survivors, low educational attainment was associated with 30% risk of breast cancer mortality (70). Adjustment for socioeconomic status accounts for much of the differences in survival for Black (74, 75) and Hispanic (66) women compared to White women. Potential mechanisms for the relation of low SES to breast cancer mortality include later stage at diagnosis (76, 77), increased risk of hormone-negative breast cancer (78, 79), and less frequent completion of high-quality treatment (1).

The interplay between socioeconomic status and race/ethnicity contributes to breast cancer disparities. Differences in utilization of early detection and treatment methods, access to quality care, and tumor characteristics contribute to the racial/ethnic and socioeconomic disparities in breast cancer mortality. Although incidence of breast cancer is decreasing in the U.S., this disparity in mortality may continue unless there is a concerted effort to introduce public policy initiatives to address broader societal inequities, which are linked to both cancer and broader health outcomes.

## Prostate Cancer

The prostate is the leading site for new cancer diagnoses and second leading cause of death from cancer among Black, White, and Hispanic men in the U.S. (1). Improvements in anti-androgen treatments and changes in the assignment of cause of death have resulted in a decrease in reported prostate cancers since 1990 (1). The only well-established risk factors for prostate cancer are age, race, and family history of the disease, with African-American men and Jamaican men of African descent having the highest prostate cancer incidence worldwide (2). The prostate cancer prevention trial showed evidence that anti-androgen therapies, specifically with finasteride, can reduce prostate cancer risk among men over the age of 55 (80).

**Figure 4:**
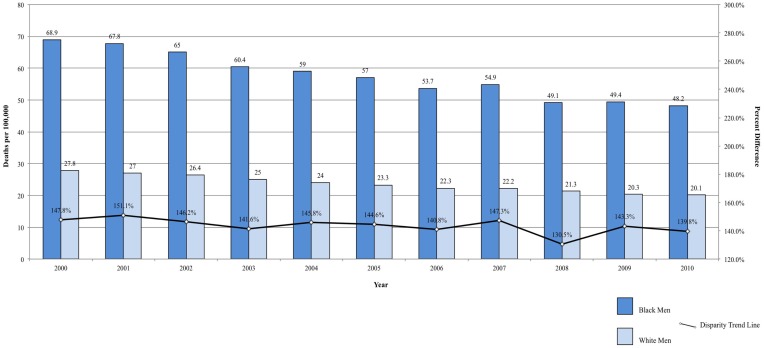
**Prostate cancer mortality among men, United States 2000–2010 and disparity ratio trend**.

In addition to significantly higher incidence of prostate cancers when compared to White and Hispanic men, Black men have the highest overall and stage-specific mortality rates (2, 81, 82). The mortality rate is almost 2.5 times higher in Black men than in White men (2). Compared to Hispanic men, the difference is even greater (37). Between 2000 and 2009, the mortality rate due to prostate cancer decreased faster among Black men compared with White men (3.7 vs. 3.4%/year), which was only slightly lower than Hispanic men (3.8%/year) (2, 37). Though Hispanics continue to have lower mortality than non-Hispanic Blacks and Whites, the steeper decline among Black men compared to White men provides evidence that disparity between the two groups is closing (2). From 2000 to 2010, the prostate cancer disparity ratio decreased from 147.84 to 139.80% higher among Black men, corresponding to a decrease of 0.80%/year in the difference between Black and White men (Figure 4). Some of the decrease in prostate cancer mortality may be attributable to improved surgical and radiologic treatment, dissemination of hormonal therapy for patients with advanced-stage disease, and early detection by prostate-specific antigen (2).

Although prognosis is generally favorable for all populations, including high 5-year survival for Black, White, and Hispanic men, studies have shown differences in treatment patterns by race, which may contribute to the higher mortality in Blacks. Data from the Cancer of the Prostate Strategic Urologic Research Endeavor Study found that Black men were less likely to receive surgery compared with White men with similar disease characteristics (83). Race was correlated with type of treatment, with Black men being less likely to have radical prostatectomy than radiation therapy or anti-androgen therapy (83). Radical prostatectomy has been associated with greater survival than other treatments, including radiation therapy and watchful waiting (84, 85). Black men are more likely to present with advanced disease, are administered different treatment regimens, have shorter progression-free survival following treatment, and have more treatment-related side effects compared to White men (86).

Like with many other cancers, socioeconomic disparities explain some of the differences in prostate cancer between races (82). Results from a large prospective study suggested an increased risk of advanced prostate cancer for a number of SES indicators, such as neighborhood deprivation (87). For high-risk prostate cancer patients, the number of bone scans, the likelihood of intent-to-treat, and the likelihood of undergoing radical prostatectomy were all greater for high SES men (88). Furthermore, overall mortality and prostate cancer-specific mortality was lower for “white collar” (high SES) men (88). These results show evidence of the underlying relationship between SES, race, and prostate cancer prognosis, since Black men are more likely to be in low SES groups. Socioeconomic factors were found to explain 15% of the difference in tumor characteristics between Black and White men, while the choice of treatment and physician explained another 17% (82). In one study, approximately 25% of the racial gap in mortality could be explained by racial differences in incidence and treatment (82).

As a result of effective early detection and treatment options, the mortality rates for prostate cancer will continue to decline for all groups. Increased access to high-quality treatment has resulted in steeper declines for Black men. Socioeconomic factors can explain some of the differences in mortality by race and may eventually lead to an elimination of prostate cancer mortality differences by race.

## Colorectal Cancer

Colorectal cancer is the second most commonly diagnosed cancer in the U.S. and the second leading cause of death from cancer for both sexes combined (1, 2). Incidence has been declining since 1985 (1). This decline may be associated with downward trend in cigarette smoking and increasing non-steroidal anti-inflammatory (NSAID) use (1). Further, colorectal cancer screening with either sigmoidoscopy or colonoscopy has been increasing since the 1990s (1).

Incidence rates for colorectal cancer are higher among Black men and women compared to White men and women (22 and 23% higher, respectively) (2). Colorectal cancer incidence is significantly lower among Hispanic men and women than among non-Hispanic Whites and Blacks (37). There are also racial differences in colorectal cancer risk by site, which may produce a difference in stage at presentation (89). Blacks are more likely to develop cancer in the colon, while Whites are more likely to develop cancer in the rectum, which is lower in the bowel and more easily detected by screening (90). Identification of pre-cancerous lesions is more difficult for cancers of the colon (90). As a result, Blacks are at increased risk of developing advanced colorectal cancer compared with Whites, and Black seniors are more likely to be diagnosed with late stage disease compared to White seniors (90, 91).

**Figure 5:**
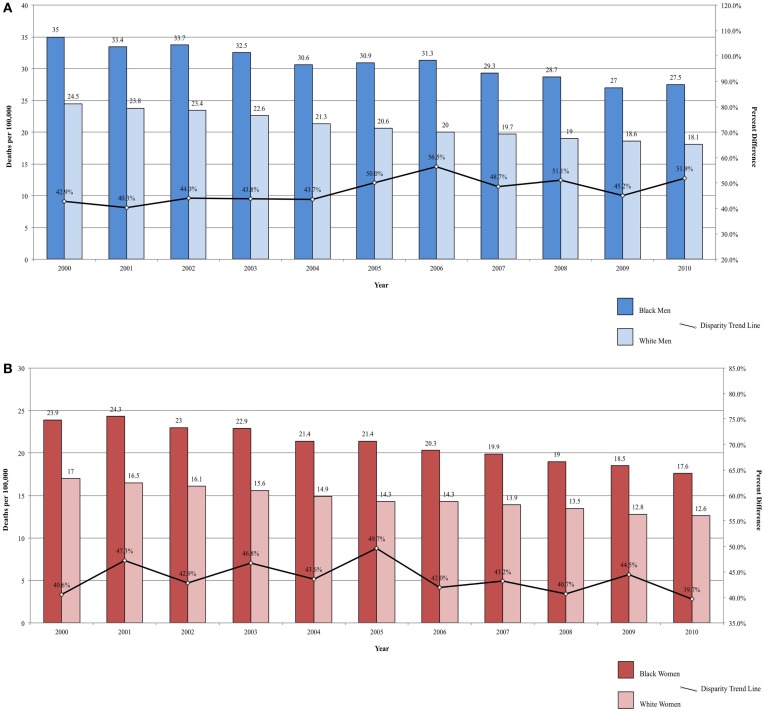
**(A)** Colorectal cancer mortality among men, United States 2000–2010 and disparity ratio trend. **(B)** Colorectal cancer mortality among women, United States 2000–2010 and disparity ratio trend.

Mortality rates are also significantly lower among Hispanic men and women compared to both Blacks and Whites (37). Mortality from colorectal cancer declined between 2000 and 2010, but the rate remains significantly higher among Blacks (34, 89, 92). Over the same time period, the disparity ratio for colorectal cancer mortality increased from 42.86 to 51.93% higher for Black men compared to White men (Figure 5A) and decreased from 40.59 to 39.68% higher for Black women compared to White women (Figure 5B). The average annual change was 0.91%/year for men and −0.09% for women. Although mortality rates are declining among both Blacks and Whites, the disparity between the two populations is not closing, and, for men, may actually be widening.

A number of factors may contribute to the disparity in colorectal cancer mortality by race, including SES, stage at diagnosis, treatment type, and physician and hospital factors (93). In a study using a micro-simulation model, 19% of the disparity in colorectal cancer mortality between Blacks and Whites could be explained by differences in screening rates and 36% of the disparity could be attributed to differences in stage-specific survival (94). In analysis of data on a large cohort of Medicare beneficiaries diagnosed with CRC, racial disparities decreased significantly after adjustment for tumor characteristics, treatment, comorbidities, hospital characteristics, and SES (93).

The relationship between race/ethnicity, SES, and their combined effect on colorectal cancer outcomes reflect the same complex relationship seen in other cancers. Comparing patients at the same stage receiving the same treatment, survival rates are more similar by race (95). A large meta-analysis showed that racial disparities in survival for colon cancer are only slightly higher in Blacks after adjusting for treatment and socioeconomic factors (96). One study found that, after adjusting for socioeconomic status, mortality in stage II and III colon cancer patients was only marginally higher among Blacks than among Whites, with a hazard ratio of 1.16 (95% CI, 1.01–1.33) (97). In another study, socioeconomic status accounted for half of the disparity in stage III of colon cancer mortality and half of the disparity in stages II and III of rectal cancer mortality (98). Together, these studies suggest that disparities in colorectal cancer mortality between Blacks and Whites can largely be accounted for by differences in treatment, hospital characteristics, socioeconomic status, and comorbidities.

The overall incidence and mortality rates of colorectal cancer have been declining since 1980s, a change that is likely attributable to reduced smoking, increased NSAID use, and widespread screening. However, Black men and women continue to have the poorest outcomes and this difference is associated with a number of societal factors. The mortality disparity may increase in coming years if SES-associated factors for Black-Americans worsen relative to White-Americans.

## Discussion

In the U.S., over the period 2000–2010, there was an observed decline in the cancer mortality rate by gender and race (Tables 2 and 3). Explanations for this improvement include decreased smoking prevalence rates, improved and increased use of cancer screenings, and more effective cancer treatments. Despite significant gains in overall cancer mortality over this time period, persistent cancer mortality disparities by race exist (Tables 2 and 3). Although a greater decline of all-site cancer mortality among Black men and women compared with their White counterparts has been observed, significant age-adjusted cancer mortality disparities by race persist. Black men and women have higher age-adjusted mortality rates across the four common cancer types reported in this review, with the exception of higher lung cancer mortality among White women than among Black women. Later stage at diagnosis and less compliance or acceptance of proffered treatment options suggested explanations for the disparities. However, why Black patients seek treatment at later stages of illness or respond differently to treatment options merits further exploration. As cancer screening rates converge, it is important to examine why racially patterned cancer survival disparities persist. An increasing body of evidence points to underlying societal inequities as a significant player in cancer outcomes disparities. How we resolve to address the underlying identified societal inequities is critical toward eliminating disparities in cancer outcomes.

**Table 2:** **Male cancer mortality rates and racial disparity ratios, United States, 2000, 2005, and 2010**.

Cancer site	Year	Black males deaths per 100,000	White males deaths per 100,000	Disparity ratio (%)
All-sites	2000	341.2	243.4	40.20
	2005	300.3	224.9	33.50
	2010	264.4	207.1	27.70
Lung	2000	100.9	75.4	33.80
	2005	87.0	69.1	25.90
	2010	73.6	59.9	22.90
Prostate	2000	68.9	27.8	147.80
	2005	57.0	23.3	144.60
	2010	48.2	20.1	139.80
Colon/rectum	2000	35.0	24.5	42.90
	2005	30.9	20.6	50.00
	2010	27.5	18.1	51.90

**Table 3:** **Female cancer mortality rates and racial disparity ratios, United States, 2000, 2005, and 2010**.

Cancer site	Year	Black females deaths per 100,000	White females deaths per 100,000	Disparity ratio (%)
All-sites	2000	193.2	166.0	16.40
	2005	180.9	156.0	16.00
	2010	166.3	145.9	14.00
Lung	2000	39.6	42.1	6.30
	2005	40.1	41.7	4.00
	2010	36.3	39.2	8.00
Breast	2000	34.4	26.2	31.30
	2005	32.8	23.5	39.60
	2010	30.2	21.3	41.80
Colon/rectum	2000	23.9	17.0	40.60
	2005	21.4	14.3	49.70
	2010	17.6	12.6	39.70

### Race, ethnicity, and socioeconomic status

A variety of data sources confirm that there are differences in cancer outcomes by race. Establishing how much of the difference in cancer outcomes is the result of societal inequities and constitutes a disparity is more nuanced. When assessing health disparities, a crucial issue lies in determining when a difference is defined as a disparity. The measurement of a disparity is complex because a disparity is not measured directly, but is rather the difference that remains after other variables have been accounted for in an analysis. The decision regarding which variables are included in an analysis will determine the magnitude, accuracy, and consistency of reported disparities across studies. Whether to adjust for income and education level, which impact health outcomes, or to consider these variables primarily the result of societal inequities and on the pathway toward health disparities, is critical to this debate. This discussion has implications for tracking disparities over time and for policy strategies to address disparities. For example, when educational attainment, a reflection of educational opportunity and quality, is viewed as a pathway variable toward health disparities, then education policy becomes a strategy to address health disparities. Policy solutions that address access to and quality of the health care system, discussed below, are certainly important toward narrowing disparities, but cannot fully redress broader societal inequities at the core of racial and ethnic health disparities.

### Patient protection and affordable care act

The U.S. has long been criticized for failing to provide universal health care access to its residents. The patient protection and affordable care act (ACA), signed into law in 2010, is designed to increase access to health insurance and high-quality care for all legal U.S. residents. Implementation of ACA will lead to a projected decrease of 12 million uninsured in 2014 and 26 million by 2017, as well as expanded health care access for millions more Americans (99). Through provision of greater access, the ACA should increase availability and utilization of preventive and treatment services, with the goal of improved health outcomes and reduced health disparities, which disproportionately impact minority populations. A number of provisions of the law address cancer-related care, including essential health benefits. The law requires all health plans to cover preventive services that receive an “A” or a “B” rating from the United States Preventive Services Task Force with no cost-sharing (100). Services covered include *BRCA* genetic counseling for high-risk patients, mammography, Pap smears, three types of colorectal cancer screening, and tobacco cessation interventions. The ACA also mandated the creation of the Patient-Centered Outcomes Research Institute (PCORI) to “… help people make informed healthcare decisions, and improve healthcare delivery and outcomes, by producing and promoting high integrity, evidence-based information that comes from research guided by patients, caregivers and the broader healthcare community” (101). Created in April 2013, the PCORI Advisory Panel on Addressing Disparities awarded a total of $52.8 million for 31 research projects – about two-thirds of which address chronic conditions, including cancer (102). While it is premature to discern the impact of ACA on overall cancer disparities, there are some early indicators of its potential.

Human papilloma virus (HPV) vaccines, which protect against strains of the virus responsible for cervical cancer, are recommended for use by the federal Advisory Committee on Immunization Practices for adolescent girls and women in 2006 and for adolescent boys and men in 2011 (103). Reported efficacies from double-blinded studies of up to 100% have been reported for HPV-16/18 bivalent vaccines against cervical cancers (104, 105). Among sexually active vaccinated girls ages 14–19, there was an 88% decrease in vaccine-type HPV prevalence in the years following the vaccination recommendations in 2006 (103). As a result of the ACA, HPV vaccination rates can be expected to increase (99) and a subsequent decline in cervical cancer incidence is predicted (106). Whether this decline is universally appreciated across racial and socioeconomic groups will need to be monitored. Cultural acceptance of this and other recommendations across communities has been mixed and merits ongoing evaluation. Prioritizing and addressing identified community concerns will be important toward achieving the high immunization and reduced cervical cancer rates across population groups.

Up to 60% of colorectal cancer deaths are estimated to be avoidable through regular colorectal screening (107, 108). Despite the proven benefits of colorectal cancer screening, only an estimated 64.5% of adults aged 50–75 years old reported being up to date with one of the three recommended screening tests (fecal occult blood testing, sigmoidoscopy, and colonoscopy) in 2008 (100). Though regular screening increased for all racial/ethnic groups between 2000 and 2008, Hispanic and Black-Americans have consistently been less likely to be up-to-date with colorectal cancer screening compared to Whites (109). The ACA’s provision of increased access to colorectal cancer screening tests through required health plan coverage and the removal of cost-sharing for fecal occult blood testing, sigmoidoscopy, and colonoscopy will likely result in a decrease in these screening disparities (110).

In 2010, 75% of women aged 40 or older reported having a mammogram within the past 2 years (95). However, after more than 20 years of increased mammography use, recent recommendation changes delay initiation of routine mammography screening until age 50 and undergoing mammography at reduced frequency (17). Higher rates of insurance and removal of cost-sharing will increase access to mammograms, and recommendation changes can be expected to better detect true disease. Breast cancer screening uptake rates with the new mammography guidelines will be known in the coming years, but long-term effects due to increased access to screening and treatment through the ACA will not be clear for much longer.

Not all of the Americans gaining insurance through the ACA will utilize preventive services. Utilization rates for recent cancer screening programs targeting vulnerable low-income populations, National Breast and Cervical Cancer Early Detection Program and the Colorectal Cancer Control Program, yielded utilization rates of 30 and 18%, respectively (111, 112). Although these programs were small and population characteristics of the newly insured U.S. population may differ, optimistic predictions on improved cancer outcomes based on high rates of screening and early disease stage detection may not be borne out and data on actual utilization of preventive services across racial and ethnic groups, even without cost-sharing, will require close monitoring.

Some apparent contradictions in ACA mandates complicate the potential positive impact of the law’s provisions on cancer care. Although the ACA mandates insurers to cover a number of preventive services without cost-sharing, it does not require insurers to cover follow-up testing when abnormalities are detected during the initial screening examination (113). Coverage for genetic counseling for women with high risk of having a *BRCA* gene mutation is mandated by the ACA, but genetic testing in these high-risk patients is not necessarily covered (113). This situation may limit potential improvements in cancer outcomes that could result from improved screening with fully covered follow-up care. Another important issue is addressing poorer outcomes among the patient populations with cancer-related comorbidities. The American Society of Clinical Oncology, in its statement on the ACA, noted that “What remains less clear is how these provisions … will affect the growing number of individuals who have already been diagnosed with cancer and are at increased risk for developing secondary cancers and other diseases …” (113).

The ACA provisions related to expanding access to preventive and early detection services will impact the number and staging of new cancer diagnoses in the coming years. It is less certain what the short- and long-term impact of the implementation of these requirements will be on overall cancer mortality and on cancer outcomes disparities. Previous health services literature suggests that the improved access to care and provider coverage requirements could accelerate the recent trend of declining cancer disparities in the U.S. The universality of improved outcomes across all population groups needs to be assured. Monitoring the magnitude and racial/ethnic distribution of outcome trends is critical. Identifying and addressing shortcomings in the legislation as it pertains to minimizing and eliminating cancer health disparities must be an ongoing process.

### Strengths and limitations

This work describes primarily Black–White differences in cancer mortality. It is important to acknowledge that data on Black-Americans cannot be assumed to be representative of other vulnerable populations. Although some of the most striking differences in cancer mortality are between Black and non-Hispanic White populations (114), there are significant disparities that affect Hispanic, Asian, and American-Indian/Alaska-Native diasporas. The topic of cancer disparities is broad, encompassing all cancer sites, and other specific sites have larger or smaller racial disparities than the sites reported here. This review focused on four major cancer sites with high incidence rates and mortality rates. These cancers; lung, colorectal, female breast, and prostate cancer, represent an estimated 50% of incident cancers and 48% of cancer deaths in the U.S. (114). Therefore, while not inclusive of specifics on all cancer types, this review highlights similarities and differences in disparity trends across some significant cancers types in the U.S.

A major strength of this review is the quality of the dataset used, the National Cancer Institute’s surveillance, epidemiology, and end results (SEER) dataset. SEER contains cancer incidence and survival data from population-based cancer registries. Dataset coverage includes approximately 28 percent of the U.S. population, including 26% of the African-American population. The representativeness of the sample and the data quality permit extrapolation to the U.S. population. Broadening the discussion framework to explore the potential impacts of ACA mandates on cancer disparities add breadth to this review’s discussion. The focus on cancer outcomes rather than incidence permits examination of disparities related to post diagnosis treatment access and care and subsequent outcomes disparities. This avoids assessing longer-term exposures, accounting for latency periods and is more reflective of disparities variables relevant to the past decade.

## Conclusion

Although progress has been made since 2002 Institute of Medicine’s report on racial/ethnic disparities, many cancer-related disparities persist. Smoking prevalence remains higher for Black men than for White men, for White women than for Black women, and among individuals of low SES. Public health programs focusing on prevention have not been universally distributed and received. Uptake of early detection methods remains low for some racial/ethnic groups. Limited access to and utilization of advanced cancer therapies persist for a number of populations. The ACA addresses these issues in a number of ways: through improved access to care, the requirement of essential benefits, and the PCORI Advisory Panel on Addressing Disparities. It is premature to predict with any certainty the impact of ACA on cancer outcomes and disparities. However, the broad reaching provisions of the law provide a measure of optimism that the law may contribute to further declines in racial/ethnic disparities in overall cancer mortality. This optimism must be tempered with awareness of the law’s limitations to redress underlying societal inequities. The implementation of the affordable care act in 2010, with its goals of addressing and further reducing health disparities, is commendable. However, it is unlikely that eliminating cancer and other health disparities can be achieved through health care mandates alone. Redefining the framework of cancer disparities research beyond health care is critical. Funding of cancer disparities research, which includes assessment of the impact of economic, education, and social policies on health outcomes, will inform and broaden the agenda toward solutions to eliminate cancer disparities.

## Conflict of Interest Statement

The authors declare that the research was conducted in the absence of any commercial or financial relationships that could be construed as a potential conflict of interest.
